# Acute Sternoclavicular Joint Sepsis With Medial Clavicle Osteomyelitis (Staphylococcus aureus) and Cervical-Thoracic Epidural Phlegmon in an Adult Female With No Apparent Risk Factors

**DOI:** 10.7759/cureus.35870

**Published:** 2023-03-07

**Authors:** Jamison K Walker, John T Cronin, Brett W Richards, John G Skedros

**Affiliations:** 1 Orthopaedics, Campbell University School of Osteopathic Medicine, Lillington, USA; 2 Orthopaedics, Utah Orthopaedic Specialists, Salt Lake City, USA; 3 Orthopaedics, The University of Utah, Salt Lake City, USA

**Keywords:** paraspinous phlegmon, medial clavicle excision, epidural phlegmon, osteomyelitis, sternoclavicular joint sepsis

## Abstract

This is a case of a 71-year-old female with a history of only one known medical problem (hypertension) who presented with a right sternoclavicular joint (SCJ) infection in addition to (1) a contiguous lower cervical and upper thoracic epidural phlegmon and (2) cellulitis and a phlegmon in her posterior neck, which was subcutaneous and near the lower cervical and upper thoracic spinous processes. These loci of infection developed several days after she had pricked her fingers when cutting rose bushes and were initially considered to be epidural abscesses. However, after the patient was transferred to our tertiary medical center, a neurosurgeon and radiologist determined that the cervicothoracic infections were phlegmons rather than fully developed abscesses. The phlegmons were treated with only IV antibiotics. The SCJ infection was surgically debrided, and the medial clavicle was excised. Bone and fluid cultures grew methicillin-sensitive *Staphylococcus aureus (S. aureus)*. The patient recovered uneventfully (the final follow-up was four years later). This case is uncommon because of the concurrent SCJ infection with medial clavicle osteomyelitis, cervical-thoracic epidural, and paraspinous phlegmons.

## Introduction

The sternoclavicular joint (SCJ) is formed by the articulation of the medial clavicle, manubrium, and costal cartilage of the first rib. The incidence of septic arthritis of the SCJ is estimated to be 2-6 out of 100,000 individuals and comprises 0.5-1.0% of all joint infections and 0.5% of joint infections in healthy patients [1-4]. Risk factors for SCJ infections include long-term immunosuppression, diabetes mellitus, IV drug use, rheumatoid arthritis, intra-articular injection, and steroid use [2,5,6]. SCJ infectious etiologies include *Staphylococcus aureus* (*S. aureus*; 44-67% of cases), *Pseudomonas aeruginosa* (*P. aeruginosa*; ~10%), *Escherichia coli* (*E. coli*; ~5%), and *Mycobacterium tuberculosis* (*M. tuberculosis*; <1%) [7,8]. Other species, such as *Streptococcus pneumonia* (*S. pneumonia*), *Streptococcus pyogenes* (*S. pyogenes*), *Clostridium perfringens* (*C. perfringens*), and other rare organisms, have also been reported [4,8,9]. Most SCJ infections are thought to originate from hematogenous spread from infections or inoculation elsewhere in the body. Diagnosing SCJ infection accurately and quickly is essential because, if left untreated, it can result in mediastinitis, retrosternal abscess, osteomyelitis, and sepsis [2,10]. Nusselt T et al. [5] concluded that when there is no bone involvement, a simple surgical debridement, and IV antibiotics are adequate. When osteomyelitis is present, surgical intervention with bone debridement is often required [2,4,11,12]. Only a few studies have reported the co-existence of SCJ infection and some form of epidural/paraspinous infection (abscess, phlegmon, and/or discitis) [13,14]. In these prior studies, the patients’ infections were in the lumbar region; we could not locate a report of a concurrent SCJ infection and a cervical-thoracic epidural abscess or phlegmon. We report the case of a 71-year-old female with no significant comorbidities who developed septic arthritis due to an SCJ infection along with epidural (deep) and paraspinous process (superficial) phlegmons of the cervical-thoracic spine region. Initially, these loci of infection in the cervicothoracic spine were considered to be epidural abscesses. However, the patient avoided surgery for these lesions because they were later determined to be phlegmons at our tertiary medical center. We report this case because of: (1) the unusual combination of phlegmons in the cervical-thoracic epidural space and cervical-thoracic paraspinous process region, and SCJ infection, in an elderly person with no apparent risk factors, and (2) the necessity of SCJ debridement with medial clavicle excision.

## Case presentation

**Figure 1:**
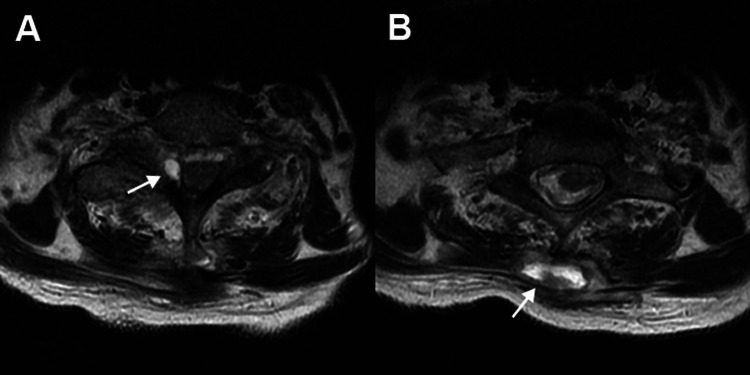
Axial MRI scan of patient's cervicothoracic spine. Axial MRI showing: (A) cervical-thoracic epidural phlegmon and (B) cervical-thoracic paraspinous process phlegmon.

**Figure 2:**
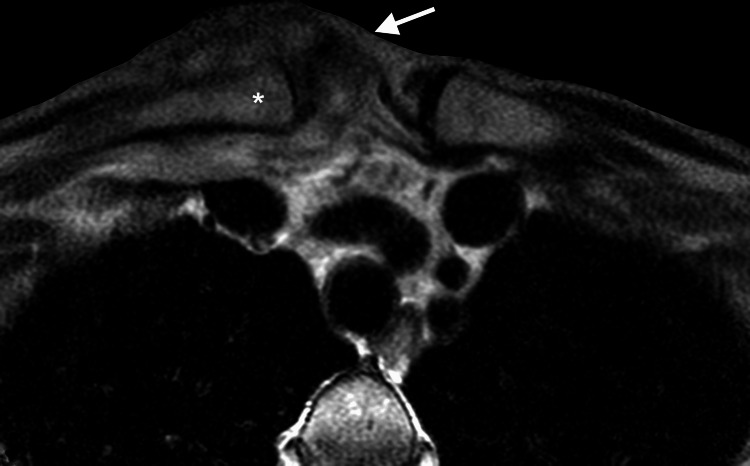
Axial MRI of patient's right sternoclavicular joint. Axial MRI scan showing the patient's right SCJ infection (arrow). The asterisk indicates the medial aspect of the clavicle, which had mild heterogeneity suggestive of acute osteomyelitis. (Motion artifact degraded the quality of this image; additional imaging was not done.) SCJ: Sternoclavicular joint.

A 71-year-old female with a history of only hypertension presented to her primary care physician (PCP) complaining of acute fever, chills, achiness, and generalized weakness. She had mild pain, swelling, erythema of her right SCJ, and a painful “lump” on the back of her neck. These symptoms developed four days after she pricked her fingers when cutting rose bushes. Initial blood tests showed elevated WBC at 16,800 cells/cu mm (normal range: 4,800-10,800 cells/cu mm), erythrocyte sedimentation rate (ESR) at 96 mm/hr (normal <20 mm/hr), and C-reactive protein (CRP) at 161 mg/L (normal <3 mg/L). She was diagnosed with cellulitis and was empirically started on oral amoxicillin/clavulanate potassium 875 mg orally twice daily, which she took for three days. She was then admitted to an outside hospital (with no consulting neurosurgeon) where MRI scanning with IV contrast of her neck and chest revealed what was believed to be at the time: (1) an epidural abscess along the right lateral thecal sac extending into the C6/C7 and C7/T1 neural foramina, (2) paravertebral abscess posterior to C6-T2 spinous processes, and (3) thickening and induration of soft tissues in and around the right SCJ, with an intra-articular effusion (Figures 1-2). Based on these MRI findings, antibiotics were switched to three grams of IV ampicillin/sulbactam every eight hours.

Due to worsening symptoms, MRI findings suggestive of an epidural abscess, and the lack of a neurosurgeon at the outside hospital, she was referred to our tertiary medical center seven days after the initial onset of symptoms. A transthoracic echocardiogram was then done and did not show evidence of cardiac involvement with respect to the infection. The ultrasonographic evaluation showed thickening of tissues around the right SCJ and fluid in the joint space. However, no fluid was obtained from the SCJ when aspiration was attempted under ultrasound guidance. Initially, it seemed likely that the patient would undergo irrigation and debridement surgery for the cervicothoracic spine infections. However, upon reviewing the MRI scan taken at the outside hospital, the consulting neurosurgeon and radiologist determined that the MRI findings were consistent with phlegmons that were likely contiguous [15,16]. Consequently, these phlegmons were treated with only IV antibiotics [17].

On the second hospital day (nine days after rose thorn pricks) at our hospital, an orthopedic surgeon (Skedros JG) performed open surgical debridement of the SCJ. A six-centimeter incision was made transversely over the SCJ. The anterior sternoclavicular ligaments were exposed and sharply divided in the transverse direction in order to gain access to the SCJ. The SCJ ligaments were carefully preserved to ensure SCJ stability. A total of 2 cc of mildly turbid fluid was found within the joint and was cultured. The synovial lining of the SCJ was then excised and sent for cultures. Then, after manually irrigating the region with saline, new surgical instruments were used, and 1.2 centimeters of the medial clavicle was resected, which would not result in SCJ instability because the insertion of the costoclavicular ligament is preserved [18,19]. Although the metaphyseal trabecular bone appeared grossly normal, a curette was used to obtain cultures. Primary closure was done over a suction drain. The fluid and bone cultures grew methicillin-sensitive S. aureus. These culture results prompted changing antibiotic treatment to 2 gms of IV cefazolin every eight hours, which was continued for six weeks [20]. This duration of treatment was also deemed sufficient for the phlegmons.

The patient recovered uneventfully. Four years after surgery, she reported no pain or discomfort with respect to the SCJ and no recurrence of infection.

## Discussion

This case is comparatively uncommon because of the concurrent SCJ infection with medial clavicle osteomyelitis and cervicothoracic phlegmons (one deep phlegmon in the epidural space and one superficial phlegmon around the spinous processes and adjacent subcutaneous tissue). Most cases of epidural abscesses/phlegmons are related to episodes of an invasion of skin flora (e.g., therapeutic injections), with *S. aureus* identified in approximately two-thirds of cases [21-23]. We only found two previously published cases that reported concurrent SCJ infection and some form of epidural/paraspinous infection (abscess, phlegmon, and/or discitis) [13,14]. Shioya N et al. [13] described a 61-year-old male with poorly controlled diabetes who developed a lumbar epidural abscess and SCJ infection from epidural injections for an L4-L5 disc herniation. The SCJ was incised, drained, and irrigated daily for one week. SCJ, blood, and urine cultures grew S. aureus, but the subsequent aspiration of the epidural abscess showed no growth. Mamarelis G et al. [14] reported the case of a 67-year-old male with a medical history of hypertension and gout that developed bilateral SCJ septic arthritis and lumbar discitis (there was no antecedent injection). Surgery and aspirations were not done for the spine or SCJ infections.

Our patient was fortunate because the cervical epidural and paraspinous infections did not progress to abscesses that would have required surgery. However, an epidural abscess was initially suspected, and we found literature concerning spinal epidural abscess to be helpful in the early management of the case [17,24]. In a recent review, Arko L et al. [24] noted that since 2000 the medical management of spinal epidural abscesses increased from 13% to 40% [21]. No studies with sufficient statistical power have been performed to accurately determine the differences in outcomes of initial surgical versus medical management [17,24]. Nevertheless, out of an abundance of caution, many authors recommend surgical intervention for epidural abscesses rather than medical management [17,24-26]. These studies are useful to remember because had our patient's infection progressed beyond the phlegmonous stage to a full epidural abscess; she would have likely required surgery.

A treatment algorithm published by Sharfman ZT et al. [17] supports the medical management of a spinal epidural phlegmon, as was done in our patient. Notably, while our patient did not require surgery since her infection did not progress past a phlegmon, she presented with five of the 13 risk factors for surgical management listed by Sharfman ZT et al. [17] (age >65, CRP >115, WBC >12.5, positive blood cultures (bacteremia), and cervical spinal level), but never experienced neurological deficits. Although our patient avoided surgery for the spine infection loci, we emphasize that healthcare providers must be diligent in monitoring patients for neurological deterioration and other factors that might herald the failure of medical management.

## Conclusions

This 71-year-old otherwise healthy female developed a right SCJ infection, a cervical-thoracic epidural phlegmon, and a phlegmon in her lower posterior neck. These loci of infection developed several days after she had pricked her fingers when cutting rose bushes. The SCJ infection required surgical debridement with excision of the medial clavicle. Bone and fluid cultures grew methicillin-sensitive *S. aureus*. IV antibiotics were given for six weeks after surgery, which treated the phlegmons and osteomyelitis of the medial clavicle. The patient recovered uneventfully (the final follow-up was four years later). This case is uncommon because of the concurrent SCJ infection with medial clavicle osteomyelitis, cervical-thoracic epidural, and paraspinous phlegmons.
